# Effect of Molecules’ Physicochemical Properties on Whey Protein/Alginate Hydrogel Rheology, Microstructure and Release Profile

**DOI:** 10.3390/pharmaceutics16020258

**Published:** 2024-02-09

**Authors:** A. Delanne-Cuménal, E. Lainé, V. Hoffart, V. Verney, G. Garrait, E. Beyssac

**Affiliations:** 1 UMR454 MEDIS, INRAE-UCA, 63000 Clermont-Ferrand, France; ameline.delanne@ucalgary.ca (A.D.-C.); ghislain.garrait@uca.fr (G.G.); eric.beyssac@uca.fr (E.B.); 2 UMR8258 CNRS—U1022 Inserm, UTCBS, Université Paris Cité, 75013 Paris, France; valerie.hoffart@parisdescartes.fr; 3 Université Clermont Auvergne, Clermont Auvergne INP, CNRS, ICCF, 63000 Clermont-Ferrand, France; pro@vincentverney.fr

**Keywords:** whey protein, alginate, molecule, cold gelation, interaction, rheology, release profile

## Abstract

The encapsulation of molecules with different physicochemical properties (theophylline, blue dextran, salicylic acid and insulin) in whey protein (WP) and alginate (ALG) microparticles (MP) for oral administration was studied. MP based on WP/ALG were prepared by a cold gelation technique and coated with WP solution after reticulation. Molecules influenced polymer solution viscosity and elasticity, resulting in differences regarding encapsulation efficiency (from 23 to 100%), MP structure and swelling (>10%) and in terms of pH tested. Molecule release was due to diffusion and/or erosion of MP and was very dependent on the substance encapsulated. All the loaded MP were successfully coated, but variation in coating thickness (from 68 to 146 µm) and function of the molecules encapsulated resulted in differences in molecule release (5 to 80% in 1 h). Gel rheology modification, due to interactions between WP, ALG, calcium and other substances, was responsible for the highlighted differences. Measuring rheologic parameters before extrusion and reticulation appeared to be one of the most important aspects to study in order to successfully develop a vector with optimal biopharmaceutical properties. Our vector seems to be more appropriate for anionic high-molecular-weight substances, leading to high viscosity and elasticity and to MP enabling gastroresistance and controlled release of molecules at intestinal pH.

## 1. Introduction

Whey protein (WP), a natural polymer well-known in the food industry because of its high nutritional value [1], was largely used for active substance encapsulation [2]. WP consists of globular proteins beta-lactoglobulin and alpha-lactoglobulin, responsible for gelling, emulsifying, foaming and hydration properties [3]. This biopolymer is able to form a cold-induced gel after a pre-heating step, which ensures WP denaturation and polymerization of WP chains by divalent cation addition [2]. This property is based on the ability of polyelectrolytes such as calcium chloride dehydrate to crosslink in the presence of counter ions to form hydrogels. The technique of encapsulation by extrusion/reticulation is simple and attractive and enables the formation of spherical particles at room temperature without the use of organic solvents, heat or vigorous agitation. In this process, the active substance can be physically or chemically entrapped and coupled with the polymers [2,4,5]. Nevertheless, WP microparticles (MP) rapidly release the active compound in simulated gastric and intestinal media, leading to nonoptimal biopharmaceutical behavior [6]. Moreover, the viscosity of the WP solution appears to be a critical point in the formation of spherical MP with controlled drug release. These limitations were partially overcome by the association of WP with alginate (ALG) to form a polymeric matrix [7,8]. Mixed WP/ALG has synergistic interactions due to repulsive and/or attractive forces between the two biopolymers [9]. In fact, ALG could form an “egg box” structure, and crosslinking for hydrogel formation occurs due to electrostatic interactions with calcium ions. The Ca^2+^ also induced whey protein cold gelation resulting from a dimeric association of guluronic acid regions with Ca^2+^ in the “egg box” formation [10]. Moreover, adding ALG to WP solution yielded pseudoplastic and viscoelastic behaviors of the gel solution [2]. Consequently, the combination of WP and ALG in the same matrix led to controlled release of MP because both polymers exhibited antagonistic behavior at an acidic pH, ALG being stably counteracted, hence the instability of WP, and, at intestinal pH, WP being stable [7]. Many parameters can affect molecule encapsulation, such as properties of polymers, formulation parameters and operating conditions [11]. Characterization and successful encapsulation can be completed by studying active substance encapsulation efficiency and parameters such as morphology, size, solubility, surface tension, thermal behavior and mechanical properties [12]. The properties of microparticles may affect the active compound release rate [11]. For example, high encapsulation of yeasts into WP/ALG MP and protection of the living cells against acidic pH and gradual release at intestinal pH validated the process of yeast encapsulation [13]. Drugs such as theophylline (THP) have also been encapsulated in WP/ALG MP [14]. Contrary to yeast, at acidic and intestinal pH, the release of TPH was rapid and the use of a WP or ALG coating was necessary to control drug release. These observations seem to indicate an influence of the encapsulated compound on the behavior of the WP/ALG MP produced. The physicochemical properties of both the polymer and drug used in the formulation can be considered to be important variables influencing solution viscosity [15,16], microparticle formation [17,18,19] and settings like encapsulation efficiency and drug/probiotic release [8,20,21,22]. Encapsulation efficiency of microparticles could also be modified by microencapsulation techniques, polymer concentration, solubility of polymers in solvent, rate of solvent removal and solubility of organic solvent in water [23,24].

In the present work, we have compared the efficiency of WP and ALG encapsulation systems to encapsulate core material (molecules) with different physicochemical properties, such as water solubility, molecular weight and ionic charge. The WP and ALG systems were compared in terms of loading/encapsulation efficiency, size distribution, shape and in vitro drug release. Four molecules, namely theophylline (THP), blue dextran (BD), salicylic acid (SA) and insulin (INS), with different water solubility (from 0.03 to 50 g/L), molecular weight (from 138 to 2 × 10^6^ g/mol) and ionic charge (positive, negative or neutral during MP formation) were selected for this purpose. The complexing of WP/ALG is pH-dependent and will take place at a pH of around 7. The solubility and charge of the selected core material will differ regarding the pH at which gelification takes place and thus affect the physicochemical nature of MP. Substance-loaded MP, coated and uncoated, were characterized in vitro. The impact of molecules on polymer viscosity and rheology was evaluated as a factor of polymer–molecule interaction. MP produced by the extrusion cold gelation of WP/ALG polymers were evaluated in terms of drug loading, encapsulation efficiency, optical microscopy, scanning electron microscopy (SEM), size, Fourier transform infrared spectroscopy (FT-IR) and in vitro release studies.

## 2. Materials and Methods

### 2.1. Chemicals

Whey protein isolate WP (Alacen^®^ 845, 93% protein content dry matter basis, NZMP, Wellington, New Zealand) and Sodium alginate ALG (Manucol^®^ DH- >99% dry matter basis, ISP, Wayne, NJ, USA) were used. Solvent (acetonitrile, methanol) and calcium chloride dihydrate (CaCl_2_), sodium hydroxide (NaOH), hydrochloric acid (HCl), sulfate sodium anhydre (Na_2_SO_4_), phosphoric acid extra pure (H_3_PO_4_) were purchased from Fisher Scientific (Illkirch, France).

### 2.2. Model Molecules

Four model molecules (theophylline, dextran blue, salicylic acid, insulin) were selected for their differences in molecular weight (from 138 to 2.106 g/mol), aqueous solubility and pKa value. Theophylline was supplied from Pierre Fabre Medicament Laboratory (Labège, France). Insulin solution was provided from Lilly France SAS. Blue dextran was purchased from Sigma-Aldrich (Saint Quentin Fallavier, France). Salicylic acid was purchased from Fisher Scientific (Illkirch, France).

### 2.3. Preparation and Characterization of Polymeric Solutions

#### 2.3.1. Stock Solution

Classically, accurate amounts of WP isolate powders were re-hydrated at room temperature in de-ionized water (1 h gentle magnetic stirring and 2 h resting) in order to prepare WP solutions (10.0 and 11.0% *w*/*w*) with complete hydration of proteins. After adjustment at pH7.0 by NaOH appropriate addition, the denaturation of proteins was inducing thermically (80 °C, 40 min [1].

ALG solutions (3.0% *w*/*w*) were prepared in de-ionized water or directly in insulin solution (for insulin loading) and stirred at room temperature.

#### 2.3.2. Molecules Added to Polymer Solution

Pure WP and pure ALG solutions were combined at 80/20 (WP/ALG *w*/*w*) ratio of WP (11.0% *w*/*w*) and ALG (3.0% *w*/*w*) and substance was added at 0.8% (*w*/*w*). The final concentrations of WP, ALG and model substance were 8.8, 2.4 and 0.8% respectively.

#### 2.3.3. Zeta Potential Measurements

Positive or negative charges of polymeric solutions were measured by laser Doppler electrophoresis using a using a Zetasizer nanoseries Nano ZS (Malvern Instruments Ltd., Malvern, UK). Data analysis of Zeta potential is presented as mean standard deviation of three samples (with ten runs for each sample).

#### 2.3.4. Viscosity Study

Evaluation of rheological properties of in vitro gelling polymers is important to predict their in vivo drug release characteristics. The spreading diameter of the hydrogels (1 ± 0.01 g, containing or not active substances) was estimated at room temperature (Brookfield digital model DVII viscometer (*n* = 3)) using two horizontal glass plates (20 × 20 cm, mass of the upper plate: 125 ± 1 g, measurement at 1 min, previously validated protocol [19,25]).

#### 2.3.5. Rheological Study

A rheological study of polymer solutions with or without CaCl_2_ (ionic gelation) was carried out using a rotating rheometer (Ares 509954812-TA Instruments). The test samples (2 g) were individually loaded on the rheometer and allowed to equilibrate at 20 °C for 5 min before the rheological measurements [26,27,28]. All experiments were performed in triplicate. The rheology of the solutions was measured with increasing or imposed stress, equipped with a cone–plane geometry (angle of the cone 0.1 radians, gap 0.048 mm, frequency 1.0 rad/s). The following protocol was applied, (i) determination of the linear viscoelastic region of each test sample by initial torque sweeps at a frequency of 1 Hz, (ii) choose of a torque value representative of the linear viscoelastic region for the frequency sweep analysis, (iii) determination of the oscillatory measurements over a frequency range from 10 to 0.01 Hz.

Most polymeric materials exhibit viscoelastic behavior, and this property can be investigated using rheological experiments such as dynamic mechanical testing, which offers a convenient way to assess time dependence of mechanical properties of polymers. The stress–strain relationship in viscoelastic material is always characterized by the complex dynamic modulus (G*) as no single parameter is able to be used. G* is resolved into two components using complex notation:

(1) $$G^{*}=\frac{\sigma }{\varepsilon }=G^{\prime }+iG^{\prime \prime }$$

G′, the storage modulus (or elastic modulus), is the real part of the complex modulus, and it describes stress–strain relationships that are in phase. G″, the loss modulus (or viscous modulus), is the imaginary component, and it characterizes the out-of-phase component. The relation between dynamic viscosity (ƞ*) and the complex modulus is provided by the following notation:

(2) $${ƞ}^{*}=\frac{\sigma }{\frac{d\varepsilon }{dt}}=\frac{G^{*}}{\left(iw\right)}={ƞ}^{\prime }-i{ƞ}^{\prime \prime }$$

with ${ƞ}^{\prime }=G^{\prime \prime }/\omega$ and ${ƞ}^{\prime \prime }=G^{\prime }/\omega$.

Finally, the real component of the complex viscosity (ƞ′) describes the viscous dissipation in the sample, while the imaginary component (ƞ″) represents the stored elastic energy. Furthermore, the tangent of the phase angle (tan δ) describes the balance between the viscous and elastic behaviors in a polymer solution:

(3) $$\tan\;\delta =G^{\prime \prime }/G^{\prime }={ƞ}^{\prime }/ƞ$$

tan δ is an indicator of the overall viscoelasticity of the sample, being a measure of the energy loss to the energy stored per cycle (G′/G″). According to the tan δ value, a solid (gel-like) response (tan δ < 1) or a liquid-like response (tan δ > 1) is obtained. A decrease in tan δ led to an increase in the elasticity of the material and a reduction in the viscous behavior.

A change in molecular parameters can be related to the evolution of the rheological material properties; thus, the linear viscoelastic properties in dynamic experiments are sensitive both to the chain scission and to the three-dimensional network formation.

Calculation of the mean dynamic moduli were performed from the 31 points of G′ and G″, measured across the frequency range. The viscoelastic nature of the test sample was also described thanks to the loss tangent, tan δ. The profile of tan δ (gel strength) was recorded at room temperature using Ta Orchestrator-7 software.

The viscoelastic properties were measured for several types of mixtures: (i) Non-gelled polymers (without CaCl_2_). The development of ƞ′, ƞ″ and tan δ was followed by frequency scanning to determine viscoelastic properties; (ii) polymers under gelling conditions (in the presence of CaCl_2_). The development of ƞ′, ƞ″ and tan δ was followed by a sweep over time, typically for 5 min at a constant frequency.

### 2.4. Microparticle Preparation and Coating

We used extrusion/cold gelation (extrusion with 23G needle, method described previously [7]) to obtain loaded WP/ALG MP. These MP were immersed in pure WP solution (10.0% *w*/*w*) maintained under magnetic stirring during 5 min and then transferred into 0.1 M CaCl_2_ solution for 1 h. The coated MP so obtained were observed microscopically.

### 2.5. Characterization of Microparticles

#### 2.5.1. Interaction of Encapsulated Substance with Reticulated Polymers by FTIR Analysis

The interaction between the different polymers and active substance composing the MP was analyzed using infrared spectrometer and accessory for attenuated total reflectance (IRAffinity-1S FTIR, Shimadzu, Kyoto, Japan and ATR-FTIR, Miracle 10). Active-substance-loaded WP/ALG MP were withdrawn, washed several times with de-ionized water and then freeze-dried. MP were frozen at −80 °C and freeze-drying was performed for 48 h in a standard freeze dryer. Frozen MP were placed in the pressure chamber (<3 Pa), maintained at −34 °C during 1 h and progressively heated to 10 °C during 24 h. During the past 24 h, temperature increased and was maintained at 25 °C. Spectra (absorbance mode 400 to 4000 cm^−1^, 200 scans at 4 cm^−1^ resolution) of active substance WP/ALG MP and active substance/WP/ALG MP were captured.

#### 2.5.2. Microparticle Size and Morphology

MP were observed optically (microscope Nikon SMZ1000, digital camera Olympus optical, Tokyo, Japan) to determine diameters of 90 MP (n = 3) by image analysis (Image Pro Plus). The network of MP extruded and lyophilized was observed under an electron microscope (MEB-FEG, University Paris Descartes). The network of MP extruded and lyophilized was observed under an electron microscope (MEB-FEG, University Paris Descartes).

#### 2.5.3. Molecules’ Encapsulation Efficiency

Encapsulation efficiency (EE, n = 3) was defined as the amount of active substance totally entrapped related to initial amount in the extruded solution volume. For TPH, BD and SA measurements were conducted spectrophotometrically at 273 nm, 618 nm and 296 nm, respectively. For INS, reverse-phase RP-HPLC MERCK-HITACHI was performed as follows: injection of 20 µL samples (autosampler AS-2000 A, pump L-62000 A, diode array detector L-45000, interface D-6000) into equilibrated (40 °C, column thermostat L-5025) LiChrospher RP-18 column (5 µm, 4 × 250 mm, Merck, Darmstadt, Germany) at a flow rate of 0.8 mL/min with 72/28 eluent A (56.8 g Na_2_SO_4_ into 2 L ultra-pure water with H_3_PO_4_ to adjust pH at 2.3) and B (acetonitrile). Absorbances were recorded at 214 nm.

#### 2.5.4. Swelling Studies of Uncoated Microparticles

Swelling of MP (2.4 g, n = 3)) was observed after 240 min of dissolution into pH 1.2 or pH 6.8 buffer (50 mL, 50 rpm, 37 °C, into Schott Duran^®^ flask), as described previously [17]. Briefly, after withdrawn, measurement of [wet diameter WD (t)] was completed on single MP. The sample mass [wet mass WM (t)] was compared to [dry mass DM (t)] obtained by drying at 60 °C. Matrix weight loss MWL (%), water uptake WU (%) and diameter changes DC (%) were obtained by calculation as follows:

(4) $$\mathrm{MWL}\;(\%)\;=\;\frac{DM\;\left(t_{0}\right)\;-\;DM\;\left(t\right)}{DM\;\left(t_{0}\right)}\times \;100$$

(5) $$WU\;(\%)\;=\;\frac{\left[WM\;\left(t\right)\;-\;DM\;\left(t\right)\right]\;-\;\left[WM\;\left(t_{0}\right)\;-\;DM\;\left(t_{0}\right)\right]}{WM\;\left(t_{0}\right)\;-\;DM\;\left(t_{0}\right)}\;\times \;100$$

(6) $$DC\;(\%)\;=\;\frac{WD\;\left(t\right)\;-\;WD\;\left(t_{0}\right)\;}{WD\;\left(t_{0}\right)}\;\times \;100$$

#### 2.5.5. Calcium Content Determination

Analysis of calcium in MP was performed by flame atomic absorption spectroscopy in an acetylene/air flame (k = 422.7 nm, bandpass: 0.5 nm, calibration curve 1–5 mg Ca^2+^/L, PU9100X; Philips Industrial & Electro-Acoustic Systems Division, Amlelo, The Netherlands) on filtered solutions of exactly weighted MP samples (n = 3), (i) suspended in water or (ii) dissolved in 3% (*w*/*v*) sodium citrate aqueous solution (calcium ion chelating agent). The calcium values reflect either the total calcium amount (ii) or the free (non-associated to alginate) calcium amount (i). The fraction of calcium associated to alginate was determined by difference between these values.

#### 2.5.6. In Vitro Dissolution Studies

Molecule releases from uncoated and coated MP (0.7 g) were performed (n = 3) in pH 1.2 (sodium chloride 0.05 M, hydrochloric acid buffer 0.13 M) or pH 6.8 buffer (potassium dihydrogen phosphate 0.05 M, sodium hydroxide 0.022 M) at 37 °C (50 mL, 50 rpm, Schott Duran^®^ flask). Samples (1 mL) were centrifuged (5000 rpm, 5 min) before determination of molecule quantities by methods described above (spectrophotometrically or using an RP-HPLC).

#### 2.5.7. Release Kinetic Model

Models of release mechanisms were achieved using the released data of the different molecules from coated and uncoated MP (in pH 1.2 and 6.8 buffers) and the Harland et al. (1988) [23] equation:

(7) $$\frac{Mt}{M\infty }=A\sqrt{t}+Bt$$

In the above equations, Mt/M∞ is the fraction of molecule released at time t; A and B are diffusion and erosion terms. As described by Harland et al., the diffusion factor prevails in the release system when A > B, while erosion predominates when A < B. The release mechanism includes both diffusion and erosion equally if A = B.

### 2.6. Statistical Analysis

All statistical analyses were conducted using GraphPad Prism 8 (GraphPad Software Inc., San Diego, CA, USA). Data are expressed as means ± Standard Error Mean (SEM). Analyses were performed using a *t*-test or a one-way ANOVA test for multiple comparisons. Differences with *p* < 0.05 were considered statistically significant.

## 3. Results and Discussion

Four model substances were selected for their differences in physicochemical properties [29,30], such as molecular weight (from low to high), aqueous solubility and pKa value (Table 1).

### 3.1. Influence of Model Molecules on Hydrogels

#### 3.1.1. Model Molecules Influenced pH and Polymeric Viscosity and Rheology

**Figure 1:**
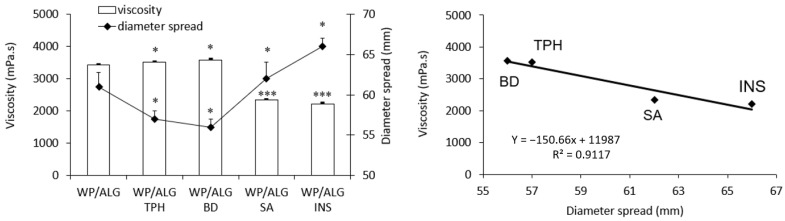
Influence of theophylline (TPH), blue dextran (BD), salicylic acid (SA) or insulin (INS) on the viscosity and the diameter spread of polymeric solutions WP/ALG. The results are presented as mean ± standard deviation (n = 3). Viscosity and diameter spread of WP/ALG TPH, WP/ALG BD, WP/ALG SA or WP/ALG INS statistically different from WP/ALG: * *p* < 0.05; *** *p* < 0.001.

**Figure 2:**
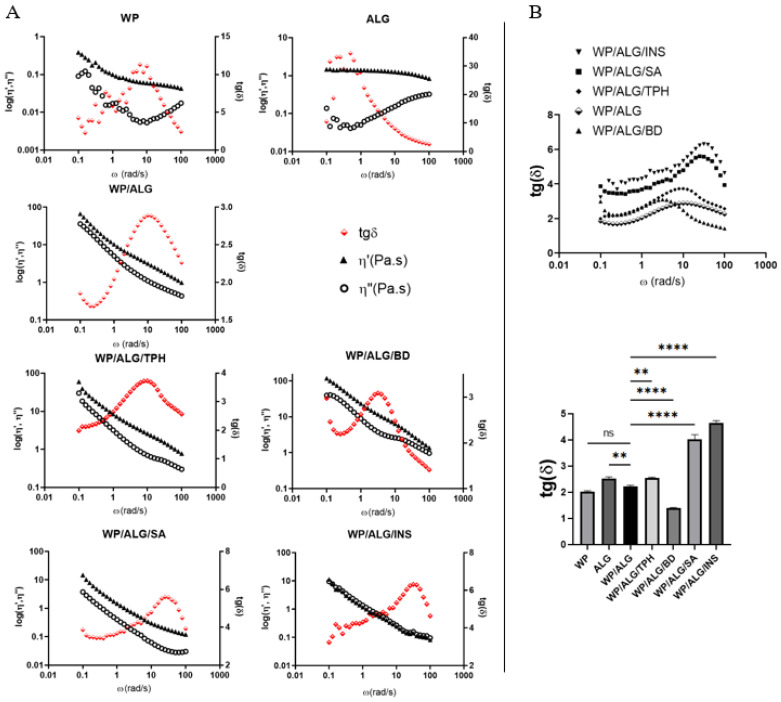
Results from the rheological measurements of pure WP, ALG, WP/ALG and WP/ALG/ substance (theophylline (TPH), blue dextran (BD), salicylic acid (SA) or insulin (INS)) as a function of frequency with (**A**) complex viscosity ƞ′ and ƞ″ (**B**) tangent delta tg (δ)–tg(δ) at 100 rad/s. Tg(δ) of WP, ALG, WP/ALG TPH, WP/ALG BD, WP/ALG SA or WP/ALG INS statistically different from WP/ALG: ** *p* < 0.01; **** *p* < 0.0001.

The analysis of viscosity/diameter spread and rheological measurements of model polymers with model molecules are reported in Figure 1 and Figure 2.

Without a substance, the WP/ALG solution had a viscosity of 3422 ± 30 mPa.s and a diameter spread of 61 ± 2 mm. As expected, addition of molecules had an influence on viscosity, which indicated an interaction between model molecules and polymers. The diameter spread of the polymeric solutions was a function of the solution viscosity (correlation coefficient of 0.9117) (Figure 1). With TPH or BD, the viscosity increased only slightly, resulting in a decrease in the diameter spread; molecules interaction with the polymeric structure seemed negligible. However, addition of SA led to a significant decrease in solution pH (from 7.0 to 4.9) and consequently to a decrease in polymeric viscosity. This could be attributed to the expansion of the polymer chain due to the intrachain electrostatic repulsion between ALG, WP and the substance [31]. With INS, the viscosity of WP/ALG was significantly decreased to 2219 ± 27 mPa.s, with an increase in the diameter spread to 66 ± 1 mm. In fact, ionic interactions with other biomacromolecules are influenced by the surface electrical properties of protein drugs such as INS [32]. The amino group and carboxylic acid group of amino acids are ionizable; thus, INS may attract positive charges due to six amino acid residues and attach negative charges via ten other amino acid residues [33]. As for SA, the intrachain electrostatic repulsion between ALG, WP and INS resulted in the expansion of the polymer chain and thus in the decrease in polymers’ viscosity.

The rheological study (Figure 2) was undertaken in order to determine if the polymers and the polymeric association exhibit gel or solution properties and if interactions between model molecules and polymers occurred.

Entrapment of molecules within the pores of the gel structure could facilitate transport and target release of drugs and bioactive compounds via controlled disassembly of the gels. The solid-like component of a viscoelastic material can be evaluated by the measurement of the energy stored and recovered per cycle of deformation through the storage modulus ƞ″ (elastic), while the liquid-like component can be estimated by the measurement of the energy lost by cycle, which is loss modulus ƞ′ (viscosity) [11,34]. For each sample, the apparent viscosity of the WP polymer decreases with increasing shear rate (Figure 2A). Presumably, an increase in shear rate led to partial destruction of the aggregated protein network structure, which reduced the resistance to flow and therefore decreased the apparent viscosity [35]. It has been previously reported that heat-induced whey protein gels are strong and rigid and have low viscoelasticity [36]. The viscosity of ALG solution decreases with increasing shear stress. Thus, the shear-thinning nature of alginate solutions at these concentrations is demonstrated as already known from previous observations [37]. Pure ALG and WP solution exhibited Newtonian flow. The polymer (WP/ALG) exhibits stronger viscosity and elasticity compared to WP or ALG polymer alone and ƞ′ and ƞ″ decrease with the frequency applied to the solution. This phenomenon showed an interaction between the protein and the polysaccharide. At neutral pH, a net negative charge may result from electrostatic interactions between proteins and anionic polysaccharides [38].

With BD in WP/ALG polymer, ƞ′ and ƞ″ decreased with a higher frequency than for WP/ALG and all the polymeric solutions. The neutral nature and high solubility of dextran, consisting of glucosyl residues linked by α (1 → 6) glucosidic bonds, lead to extensive utilization of this polysaccharide for glycation of proteins [39,40]. Its high molecular weight increased viscosity and elasticity, suggesting that the presence of dextran in the WP/ALG matrix provided a reinforcement effect. The presence of TPH in polymers did not weaken the gel matrix, which indicates that a firm gel is formed between WP and TPH due to their synergistic interactions. Addition of SA or INS in polymers led to a significant decrease in ƞ′ and ƞ″. This decrease in viscosity and elasticity indicated a looser structure constituted by both polymers. SA and INS were mainly negatively charged and thus repulsed with ALG and WP negative charges. This could be attributed to the expansion of the polymer chain due to the intrachain electrostatic repulsion between ALG, WP and substances [31].

In addition, the loss tangent, tan δ, was determined; it is considered as another useful parameter summarizing the rheological semisolids [16,41]. tan δ is an indicator of the overall viscoelasticity of the sample, being a measure of the energy loss to the energy stored per cycle (G′/G″). tan δ < 1 indicates a solid (gel-like) response, whereas tan δ > 1 reflects a liquid-like response. Thus, as tan δ becomes smaller, the elasticity of the material increases, whilst the viscous behavior is reduced. tan δ of all polymeric solutions is very weak (<5) at high frequency. Polymeric solutions are a viscoelastic solid with ƞ′ > ƞ″. This is due to links inside the material, for example chemical bonds or physical–chemical interactions. The same results were observed by L. Nwokocha and PA Williams [42]. In summary, all the WP/ALG solutions can be described as weak gel-like systems because ƞ′ and ƞ″ were almost parallel, ƞ″ > ƞ′ and both moduli varied with frequency [43,44,45]. The polymeric interactions between WP/ALG and the substances are stronger for BD > TPH > SA > INS.

#### 3.1.2. Model Molecules Did Not Influence Functional Groups of the Polymeric Solution

**Figure 3:**
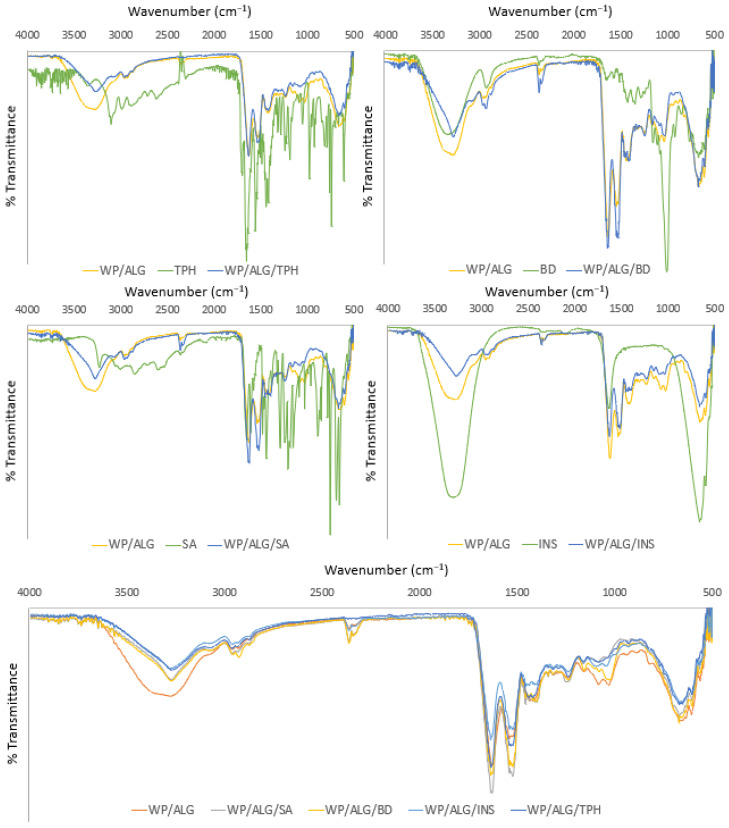
Infrared spectra of model molecule alone (SA, BD, INS and TPH), WP/ALG solution and model molecule with WP/ALG.

IR spectroscopy was used as a tool to detect incompatibility between substances and polymers. Figure 3 shows the IR spectra of the pure substance, pure WP/ALG and the combination formulation. FTIR showed many intense, sharp absorption peaks that are due to the different functional groups present in the molecules.

The spectrum of WP/ALG presented five major absorption peaks from (i) WP in the range of 1200–1350 cm^−1^, related to combination of N-H in-plane bending with C-N stretching vibrations (amide III); 1400–1550 cm^−1^, associated with N-H bending (amide II); 1600–1700 cm^−1^, governed by stretching vibration of C=O and C-N groups (amide I); 2850–2980 cm^−1^, assigned to symmetric and asymmetric C-H stretching vibrations; and 3000–3600 cm^−1^, attributed to free and bound O-H and N-H groups [46], (i) AlG with a very intense band at 1600 cm^−1^ relating to the asymmetric elongation of the carboxylate (COO^−^), which confirms the high uronic acid content of these biopolymers. The band at 1415 cm^−1^ is attributed to C–OH strain vibration and symmetrical group strain (COO^−^) vibration. ALG also exhibits, at 1030 cm^−1^ a band corresponding to the CO group [47].

TPH presented a peak at wave number 3120.26 cm^−1^, which shows the –NH stretching; the wavelengths of 2919.7 cm^−1^ and 2842.56 cm^−1^ show the presence of methyl groups, and wavelength 1666.2 cm^−1^ indicates the presence of a C=O amide group. The dextran presented a broad peak at the region of 3500 cm^−1^, corresponding to OH stretching vibration. Similarly, the peak at 2920 cm^−1^ was assigned to C–H stretching vibrations of dextran. The sharp peak at 1003 cm^−1^ and peak at 1143 cm^−1^ represent the characteristic band of asymmetrical C–O–C vibrations. Small shoulder peaks at 816 and 920 cm^−1^ confirm the presence of (1→3)-α-d-glucan, a ring structure of glucose molecules. The characteristic bands of pure salicylic acid functional groups are observed in the FTIR spectra: hydroxyl group, –OH of –COOH, at 3237 cm^−1^; C–H bond at 2861 cm^−1^; carbonyl group, C=O of –COOH, at 1658 cm^−1^; C=C double bonds of the aromatic ring at 1612 cm^−1^ and 1578 cm^−1^; phenolic hydroxyl group, –OH of the aromatic ring, at 1325 cm^−1^; simple C–O bond of –COOH at 1295 cm^−1^; and unsaturated =C–H at 760 cm^−1^ and 698 cm^−1^. Additionally, the FTIR spectra of pure salicylic acid reveal some peaks in the range 1444–1484 cm^−1^, attributed to the characteristic vibration of the C–C simple bond, and in the range 1190–1249 cm^−1^, attributed to the characteristic vibration of the phenolic C–OH bond. The FT-IR spectrum of insulin was found to have two characteristic absorption peaks, one at 1664 cm^−1^ for amide I and another at 1531.63 cm^−1^ corresponding to amide II, mainly due to C=O stretching vibration characteristic of protein spectrum. The peaks obtained with WP/ALG and model molecules revealed that there was structural integrity of both polymers and molecules. No alteration was observed in the functional groups of the substances and polymers. Thus, no strong interactions between polymer and substance were suspected (Figure 3). Consequently, configurations of the functional groups present in both WP/ALG and the model substances were unmodified and the efficient chemical stability was evidenced. However, as the ratio is predominant in WP (ratio 80/20 *w*/*w*), WP is therefore logically found in the majority.

### 3.2. Influence of Model Molecules on Microparticle and Coating Formation

#### 3.2.1. Model Molecules Influenced Reticulation Step

**Figure 4:**
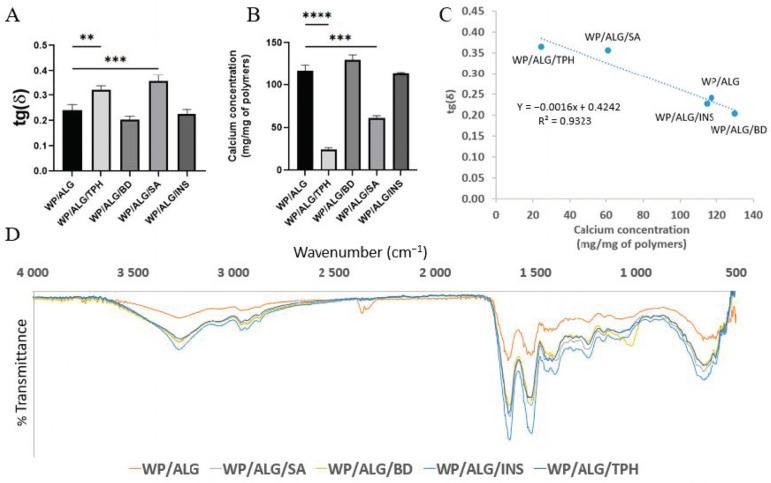
Effect of molecule incorporation (theophylline (TPH), blue dextran (BD), salicylic acid (SA) or insulin (INS)) into MP (made of WP/ALG) on (**A**) the rheological measurements tg(δ) after 5 min of calcium reticulation and (**B**) the crosslinking between polymers and calcium used for reticulation. TPH-MP and SA-MP statistically different from unloaded MP (MP). tg(δ) and calcium concentration of WP/ALG TPH, WP/ALG BD, WP/ALG SA or WP/ALG INS statistically different from WP/ALG: ** *p* < 0.01; *** *p* < 0.001, **** *p* < 0.0001 (**C**) correlation between tg(δ) and calcium crosslinking. (**D**) Infrared spectra of model molecules encapsulated in WP/ALG MP (reticulation of polymer containing molecule in CaCl_2_).

The physicochemical properties of the incorporated molecules had an impact on polymeric viscosities, indicating an interaction between molecules and polymers. These interactions can potentially modify the capacity of polymers to crosslink with calcium. To evaluate the influence of substances on crosslinking, a rheological study (Figure 4A) after 5 min of calcium reticulation and the amount of calcium crosslinked in the polymeric matrix (Figure 4B) was conducted by flame atomic absorption spectroscopy.

tan(δ) represents the state of the gel samples, and the tan δ values in Figure 4A were consistently less than 1, indicating that the gels exhibited elastic characteristics after calcium reticulation. The gels were very strong, with calcium reticulation whatever the substance loaded.

With unloaded MP, approximately 15 µg of calcium was linked to 1 mg of polymers. These results confirmed calcium’s ability to interact with ALG and WP polymers. In fact, calcium has two positive charges and can therefore attract/bond to two of the negatively charged ions on the ALG. Cationic Ca^2+^ can bind to the anionic groups on the surfaces of the protein molecules and neutralize the net negative charge, thereby reducing the electrostatic repulsion between the protein molecules and allowing them to come closer together (Figure 4B–D). Moreover, cationic Ca^2+^ can act as electrostatic bridges between anionic groups on two protein molecules, thereby linking them together [48]. The participation of hydroxyl and carboxylate groups of ALG in the calcium formed a chelating structure [49]. The interaction sites between calcium and WP are the carboxyl oxygen and amino nitrogen atoms. These changes in bands indicate that some bonds in the WP bind with calcium ions [50]. BD was anionic and consequently did not interfere in the crosslinking between polymers and calcium. As for BD, the negatively charged INS did not seem to interfere in the crosslinking between polymers and calcium, or at least the amount of calcium linked to the matrix structure remained constant. The addition of TPH and SA provided a significant decrease in the associated calcium amount. TPH was positively charged during MP formation and could interact with WP and/or ALG, resulting in less linkage between calcium and WP or ALG. For SA, which was negatively charged, competition between SA and polymers could occur for calcium interaction. These interactions between all the components were reported by other authors [22,51,52,53]. Moreover, the number of negative charges regarding WP chains decreased in accordance with the addition of the acidic SA via a decrease in pH. Thus, the amount of calcium that can be engaged with WP chains decreased. Meanwhile, the tang(δ) of gels was correlated (superior to 0.9) with the calcium concentration in the gel (Figure 4C), suggesting that calcium linked to the polymers enhanced the elastic properties of gels. From rheology and calcium binding results, we can assume that BD- and INS-loaded MP will be dense and reticulated as unloaded MP, while SA-MP will be dense but partially reticulated. TPH-MP will be less dense and reticulated than unloaded.

tan(δ) corresponded to the strength of the network structure. The presence of calcium in MP increased, indicating that it was conducive to the construction of a strong gel network structure [54].

**Figure 5:**
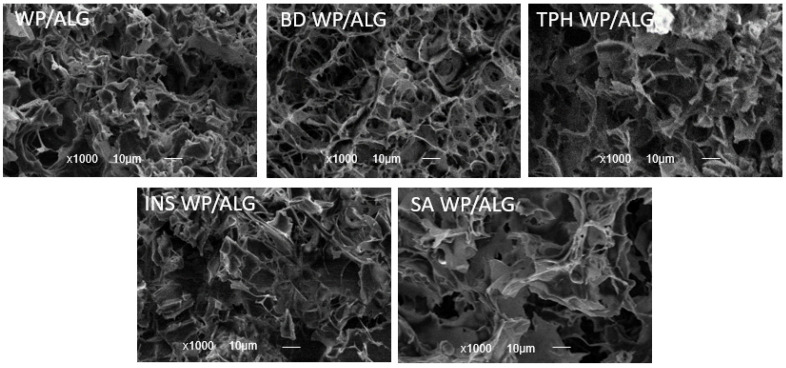
Observation of the morphology of WP/ALG MP after substance incorporation (theophylline (TPH), blue dextran (BD), salicylic acid (SA) or insulin (INS)) in electron microscopy at ×1000 magnification.

The morphology of the MP was analyzed using SEM (Figure 5). WP/ALG MP appeared to consist of a highly aggregated network of relatively small protein lumps with some large pores. The surface of MP was rough, containing a large amount of porous structures and water channels. BD and INS MP presented the same WP/ALG MP morphology. However, TPH and SA modified the structural integrity of the WP/ALG gel. As a result, the calcium concentration and gel strength of MP were correspondingly lower, which agreed with the above analysis. Compared with WP/ALG MP, TPH and SA MP had a less uniformly distributed porous structure and an increasing number of water channels. The network structure of TPH and SA MP became less dense and the pore diameter increased, which might be related to the formation of the WP/ALG network structure and the establishment of a heterogeneous network structure [55].

#### 3.2.2. Model Molecules Influenced Encapsulation Efficiency and WP Coating

**Figure 6:**
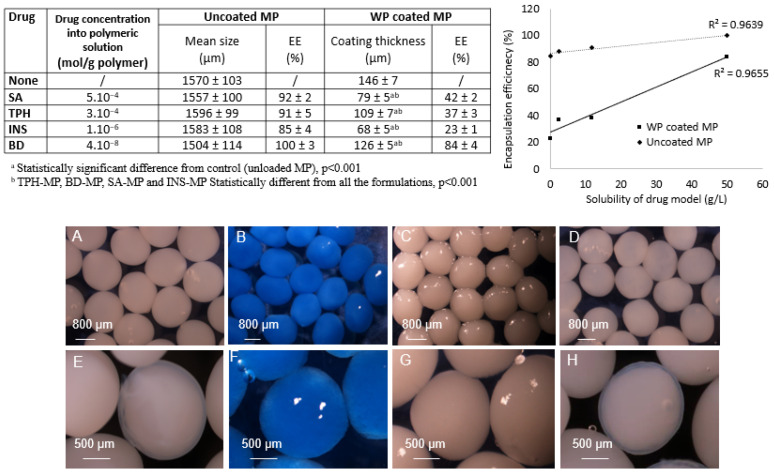
Determination of the molecule concentration in polymeric solution (mL/g of polymer), mean size of uncoated MP (µm), WP coating thickness and percentage of molecules encapsulated (EE, %) with or without WP coating. Relation between molecule model encapsulation efficiency (EE, %) and molecule model solubility (g/L): uncoated WP/ALG MP (♦) and WP-coated WP/ALG MP (■). R^2^ represents correlation coefficient (n = 3). Photographs of uncoated (**A**) theophylline-loaded MP (TPH-MP), (**B**) blue-dextran-loaded MP (BD-MP), (**C**) salicylic-acid-loaded MP (SA-MP), (**D**) insulin-loaded MP (INS-MP) and WP-coated (**E**) TPH-MP, (**F**) BD-MP, (**G**) SA-MP, (**H**) INS-MP.

MP were realized by the extrusion/cold gelation technique of WP/ALG hydrogel using standardized and reproducible conditions [17]. The type of molecules had no impact on MP morphology because substances were incorporated in MP at a percentage where the viscosity of all the polymers was preliminarily determined and permitted the formation of spherical beads for all formulations (Figure 6). Indeed, molecules’ physicochemical properties influenced the viscosity of the polymeric solution, which is a crucial parameter for microparticle production: a specific viscosity is required to allow extrusion and reticulation of spherical microparticles [17]. The spherical MP obtained had a diameter of approximately 1.5 mm for all the formulations (Figure 6).

The encapsulation efficiency (EE) parameter was appreciated as an indicator of suitability for the substance formulation process. A linear relationship was obtained between EE and solubility of molecular model. The maximum molecule content in the polymeric solution seemed to increase with molecular solubility. The EE values are relatively high, with 100% value for the high-molecular-weight uncharged BD, approximately 90% for the soluble TPH and SA and approximately 85% for INS. BD was totally encapsulated, indicating entrapment in the polymer network due to high molecular weight. On the contrary, TPH, a highly soluble molecule with low molecular weight, diffused outside MP into a calcium bath during the MP formation. The solubility of TPH was higher than that of SA, but no significant difference regarding EE was found between these two small compounds. Despite a high molecular weight (approximately 5800 Da), INS had the lowest EE. This could be explained by the strong repulsion occurring between the negatively charged INS and polymer during MP formation. Hydrogen bonding or trapping strength inside beads could be changed by the structure (ionic charge) and solubility of molecules [56].

All the investigated formulations were successfully coated. Increasing the thickness of the polymer film is important to decrease the porosity of the film coating, therefore retarding drug release [57,58]. The coating thickness of unloaded MP was estimated to be approximately 150 µm. The thickness of the coating decreased systematically when a substance was encapsulated: the physicochemical properties of the molecule impacted the coating thickness. Ionic interaction between the WP coating and the substance in the MP could be responsible for this decrease. In fact, TPH was positively charged during MP formation and thus interacted with negatively charged of WP and ALG, which were probably less linked with calcium. Consequently, fewer calcium ions were present at the surface of the MP and the negative charges of the WP coating were less linked, resulting in a thinner coating. The same results were observed with SA and INS, which were negatively charged and thus repulsed with negative charges of WP coating. A strong repulsion of the polymers could occur, and thus the coating was unstable. For BD, an anionic molecule during MP formation, the decrease in coating thickness could be due to the high molecular weight of the molecule, which can disturb the polymer network or create steric encumberment, limiting interactions between the polymeric chain and calcium ions. The coating step had a drastic consequence on EE for TPH, SA and INS, indicating loss of drug by diffusion out of the MP [14]. The diffusion of the molecules was favored either by low molecular weight or ionic repulsion.

### 3.3. Influence of Model Molecules on Controlled Release

**Figure 7:**
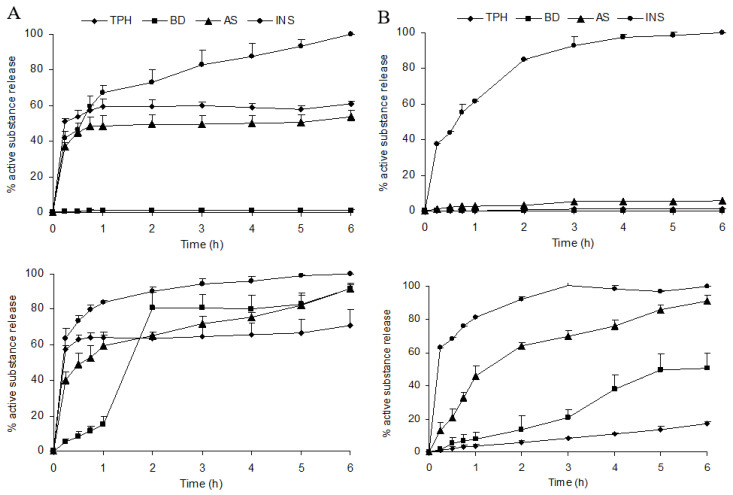
In vitro release profiles of (**A**) uncoated or (**B**) WP-coated MP loaded in theophylline (TPH), blue dextran (BD), salicylic acid (AS) or insulin (INS). Experiments were performed in hydrochloric acid at pH 1.2 (**top**) or phosphate buffer at pH 6.8 (**bottom**) at 37 °C. The results are presented as mean ± standard deviation (n = 3).

The release of encapsulated molecules from WP/ALG MP can be affected by physicochemical parameters such as molecule solubility and therefore composition of dissolution buffer pH [59,60]. Release of molecules from uncoated or coated MP was evaluated by using a classical in vitro dissolution method. Release profiles in both gastric (pH 1.2) and intestinal (pH 6.8) pH were determined over a period of 6 h (Figure 7, Table 2).

**Figure 8:**
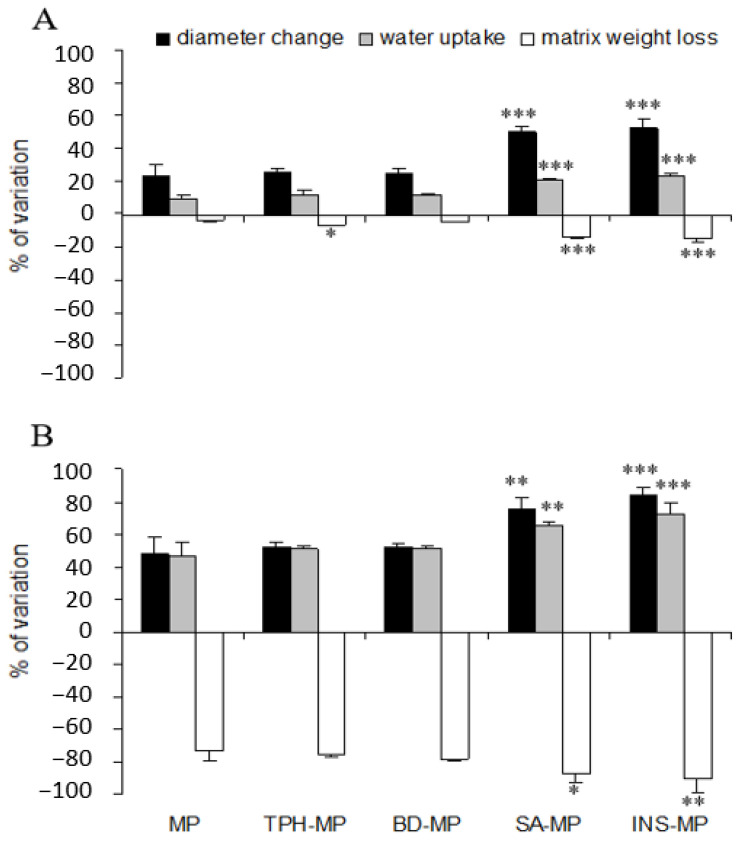
Evaluation of the swelling behavior of unloaded MP (MP) or loaded in theophylline (TPH-MP), blue dextran (BD-MP), salicylic acid (SA-MP) or insulin (INS-MP) during 240 min of dissolution at (**A**) pH 1.2 and (**B**) pH 6.8. Diameter change (mean ± standard deviation, n = 90) reflects the MP swelling evaluated by size and weight measurements, water uptake and matrix weight loss (mean ± standard deviation, n = 6). TPH-MP, BD-MP, SA-MP and INS-MP statistically different from MP: * *p* < 0.05; ** *p* < 0.01; *** *p* < 0.001.

In pH 1.2 buffer, no release was observed from uncoated BD-MP: MP were gastroresistant. A burst release of approximately 35 to 50% was observed in 15 min for the other molecules. The release stayed at a level incomplete for TPH and SA, whereas INS release plateaued. According to the Harland equation, the release of TPH, SA as well as INS was due to drug diffusion (correlation coefficient r between 0.672 and 0.987). The MP seemed visually still intact without any visible degradation after 2 h. At intestinal buffer (pH 6.8), TPH, SA and INS were rapidly released from MP due to diffusion of the substance emphasized by polymer degradation [61,62,63]. For BD, the release was mainly due to erosion (B > A) of the MP. Differences in release patterns were observed when MP were coated with pure WP solution. The WP coating layer prevented molecules’ release in acidic pH but not for INS. Ionic repulsion between INS and WP probably limited the anchor of the coating layer and thus the coating efficacy. In pH 6.8 buffer, the INS release was fast and complete without the benefit of coating. For other molecules, WP coating led to controlled release. Swelling studies were realized in order to better understand the impact of molecules on the physical characteristics of MP and consequently on release behavior (Figure 8).

As already described for unloaded WP/ALG MP [13], after 240 min at pH 1.2, few increases in diameter (23%), water uptake (10%) and matrix weight loss (4%) were observed. Indeed, matrix shrinkage is favored by electrostatic repulsions of protonated carboxyl groups of ALG with very low acidic pH [15,64]. On the contrary, matrix relaxation occurred with positively charged WP chains (isoelectric point of 5.2) repelling cations binding polymer chains (Ca^2+^) [65,66]. Combining in the same matrix WP and ALG generated low swelling behavior with little matrix degradation. At pH 6.8, even if WP is known to be stable [65], fast increases in water uptake and diameter change occurred: MP are rapidly degraded. The common explanation is the high sensitivity of ALG to intestinal pH [67] due to already demonstrated exchange of Ca^2+^ ions binding to ALG carboxyl groups and phosphate ions of the medium [64,66].

Whatever the pH, encapsulation of TPH and BD had no effect on MP swelling, whereas SA and INS increased water uptake, diameter change and matrix weight loss significantly. This increase was probably due to the ionic repulsion between the substances and polymers. These results explained the fast release of SA and INS from MP at gastric and intestinal pH. The release of SA was due to drug diffusion from the matrix because of a low molecular weight, and this release was accelerated by ionic repulsion. INS, a high-molecular-weight molecule, emphasized the swelling and destructuring of the polymer network, probably by interaction with the matrix. When the matrix swells, the relative polymer concentration decreases due to hydration and dilution [66]. This decreases the viscosity, thereby reducing the viscous force opposing liquid uptake. The balance of forces promoting water entry and the viscous force opposing it determine the rate of water uptake into the polymer beads and thus the diffusion of the molecules [2,66].

From a release point of view, WP/ALG MP led to controlled release for anionic and high-molecular-weight molecules; this controlled release being reinforced by a coating layer. Indeed, for anionic molecules of high molecular weight such as BD, at gastric pH, the swelling of the MP was low and permitted a gastroresistance of the MP. At intestinal pH, BD was release thanks to the swelling and the erosion of the MP. For soluble molecules with low molecular weight such as TPH, a diffusion of drug from MP was observed with a rapid release at pH 1.2 and 6.8. However, WP coating ensure MP gastroresistance and controlled release over 6 h at intestinal pH. For negatively charge, small molecules such as SA, controlled release can be obtained after coating. Unfortunately, for negative charge, high molecule such as INS, rapid release cannot be avoided.

## 4. Conclusions

Encapsulation is an effective tool for delivery of sensitive substances such as proteins or to control drug delivery. It has been exploited as a technique that can protect drugs from gastric pH and gastric and intestinal enzymes. One encapsulation approach is based on barriers made from natural polymers. For effective encapsulation, optimal manufacturing has to be ensured. In this paper, the desired polymer concentration and preferred molecule release profile were dependent on the physicochemical properties of the encapsulated material.

The benefit of molecules’ encapsulation into WP/ALG MP can be considered regarding two different aspects: encapsulation efficiency and controlled release. Different molecules with different physicochemical properties were successfully encapsulated into WP/ALG MP with high encapsulation efficiency. The release profiles were dependent on the substance encapsulated.

The decrease in polymer viscosity following the addition of a substance as measured before reticulation resulted in a decrease in MP resistance even if reticulation was not always impacted. This decrease was correlated with MP swelling and consequently with diffusion of molecules from the matrix for the different pH values tested. The measured rheologic parameters before extrusion and reticulation appeared to be important to study. In fact, it would enable optimizing the drug release profile by controlling reticulation degree and coating addition. The physicochemical properties of encapsulated molecules had an impact on the viscosity and elasticity of the polymeric solutions, leading to an increase in MP swelling and to fast release of molecules. Thus, the higher the viscosity and elasticity of a hydrogel, the more resistant the gel will be to diffusion, resulting in a diffusion-controlled release mechanism for molecules.

To conclude, uncoated and coated WP/ALG MP could be an interesting vector for oral administration of anionic macromolecules by providing high-viscosity hydrogels and consequently maximum gastroresistance and controlled release at intestinal pH.

## Figures and Tables

**Table 1:** Physicochemical properties of the four model substances: theophylline, blue dextran, salicylic acid and insulin.

Properties	None	Theophylline C_7_H_8_N_4_O_2_	Blue Dextran (C_6_H_10_O_5_)n	Salicylic Acid C_7_H_6_O_3_	Insulin C_257_H_383_N_65_O_77_S_6_
Molecular weight (g/mol)	-	180	2 × 10^6^	138	5807
Solubility (g/L) in water	-	11.8	50	2.5	0.0347
pKa	-	8.81	Neutral	3.0	5.4
polymeric solutions pH	7.0	7.1	6.9	4.9	6.9
Ionic charge of molecule during MP formation	-	+	+	-	-
Zeta Potentials (mV) of polymeric solution	−70	−66	−64	−32	−65

**Table 2:** Influence of physicochemical properties of molecules loaded (TPH, BD, SA or INS) and of WP coating on substances’ release mechanism (diffusion or erosion) from MP in pH 1.2 and pH 6.8 buffers, fitted to equation proposed by Harland.

Dissolution Medium	Formulation	Harland Equation		Correlation Coefficient	Mechanism of Release
		A	B	r	
pH 1.2 buffer	TPH	0.691	0.000	0.987	Diffusion
	BD	0.006	0.000	0.672	Diffusion
	SA	0.563	0.000	0.936	Diffusion
	INS	0.516	0.000	0.971	Diffusion
	WP-coated TPH	0.000	0.002	0.950	Erosion
	WP-coated BD	0.000	0.000	0.809	No release
	WP-coated SA	0.451	0.000	0.970	Diffusion
	WP-coated INS	0.551	0.000	0.986	Diffusion
pH 6.8 buffer	TPH	0.771	0.000	0.876	Diffusion
	BD	0.000	0.324	0.952	Erosion
	SA	0.416	0.000	0.988	Diffusion
	INS	0.810	0.000	0.962	Diffusion
	WP-coated TPH	0.008	0.024	0.996	Erosion
	WP-coated BD	0.000	0.091	0.982	Erosion
	WP-coated SA	0.389	0.000	0.982	Diffusion
	WP-coated INS	0.485	0.000	0.993	Diffusion

## Data Availability

All generated data are included in this article.
